# Chinese Herbal Medicine and Depression: The Research Evidence

**DOI:** 10.1155/2013/739716

**Published:** 2013-02-10

**Authors:** Lee Butler, Karen Pilkington

**Affiliations:** ^1^Department of Complementary Medicine, School of Life Sciences, University of Westminster, London W1W 6UW, UK; ^2^Department of Human and Health Sciences, School of Life Sciences, University of Westminster, 115 New Cavendish Street, London W1W 6UW, UK

## Abstract

*Background*. Alternative approaches for managing depression are often sought and herbal mixtures are widely used in China. The aim of this paper was to provide an overall picture of the current evidence by analysing published systematic reviews and presenting a supplementary systematic review of trials in Western databases. *Methods*. Searches were conducted using AMED, Cochrane Library, EMBASE, MEDLINE/PubMed, PsycINFO, and trial registers. Results were screened and selected trials were evaluated by two reviewers working independently. Systematic reviews were identified and assessed using key criteria. *Results*. Five systematic reviews were located addressing the Chinese literature, adjunctive use of Chinese herbs, and the formulae Chaihu-Shugan-San, Xiao Yao San, and Free and Easy Wanderer Plus. The supplementary review located 8 trials, 3 of which were not included in previous reviews. Positive results were reported: no significant differences from medication, greater effect than medication or placebo, reduced adverse event rates when combined or compared with antidepressants. However, limitations in methodology and reporting were revealed. *Conclusions*. Despite promising results, particularly for Xiao Yao San and its modifications, the effectiveness of Chinese herbal medicine in depression could not be fully substantiated based on current evidence. Further well-designed, well-reported trials that reflect practice may be worth pursuing.

## 1. Introduction

Knowledge about the use of complementary therapies by people with mental illness is increasing. Previous studies have demonstrated that psychiatric disorders are common among those seeking to use complementary and alternative medicine (CAM) and that anxiety and depression are among the most common reasons for people to seek care from complementary practitioners [1–4]. Over 50% of respondents self-diagnosed with “anxiety attacks” or “severe depression” reported using complementary and alternative therapies to treat their condition [2]. A recent survey in England also found that presence of anxiety or depression was an independent predictor of complementary therapy use [5]. People with mental illness appear to derive a broad range of benefits from complementary practices and these include enhancement of social, spiritual, general, and self-functioning [6]. One survey of women with depression revealed active seeking for treatments based on “natural approaches” and for those that match the individual's own values and beliefs [7]. People with depression may also be seeking to avoid adverse effects associated with conventional medication. However, CAM use has been found to be common even in those taking prescription drugs [5].

Kessler et al. [2] found that use of herbs or diet supplements was particularly prevalent in those with psychiatric problems. Use of CAM in the USA increased by 2% between 2002 and 2007 [8] with natural products reported to be the most frequently used therapies. Similar increases have been observed in Australia [9] while use in the UK continues to be substantial [5]. Users were found to be more likely to have received mental health and primary care treatment and to be dissatisfied with their overall healthcare than nonusers [10]. Chinese herbal medicines were not specifically mentioned, but several of the herbs listed are included in Chinese herbal formulae. The UK survey also revealed frequent use of herbs but limited reported use specifically of Chinese herbs [5]. In contrast, 7% of participants in a national survey in Australia reported using Chinese herbal medicine, of whom 32.9% had visited a Chinese herbal medicine practitioner [11]. 

It is difficult to fully assess the extent of use of Chinese herbs in depression outside China. Chinese herbs may be less likely to be reported in surveys as participants may be unable to name the specific herbs that they have received. Additionally, in contrast to “Western” herbs, access to Chinese herbs is generally via consultation with a practitioner rather than purchase over the counter which limits their use somewhat. Patterns of use may also vary according to ethnic group and be higher in those who consider Chinese medicine to be their primary medical system. For example, use of herbs was shown to be greater in Chinese and Japanese women in midlife than in Whites and African Americans [12]. Considerable promotion of the benefits of Chinese herbs is also found on the internet suggesting that use may be more widespread than is apparent from surveys. 

Numerous systematic reviews and research papers have been published on the effects of Western herbs such as St John's wort (*Hypericum perforatum*) in the treatment of depression (e.g., [13–15]). Chinese herbs appear to have received less attention although there has been research interest in one specific formula, Xiao Yao San. Systematic reviews of the evidence on Chinese herbs in depression have been published previously but none has addressed all RCTs of Chinese herbs in the Western literature.

## 2. Aim

The aim of this paper was to assess the evidence on Chinese herbal medicine treatments for depression based on previous systematic reviews plus a supplementary systematic review of trials in the Western literature. A secondary aim was to explore the proportion of studies on this topic that can be found in Western databases.

## 3. Materials and Methods

### 3.1. Searches

Randomised controlled trials (RCTs) of the treatment of depression with Chinese herbal medicine were located by searching AMED, EMBASE, MEDLINE, PsycINFO, PubMed, Cochrane Field for Complementary Medicine website, and the Clinical Trials Register of the Cochrane Collaboration Depression, Anxiety & Neurosis Group (CCDANTR). Only trials published in English were included at this stage. In the second stage, more comprehensive searches were conducted and trials in all languages were included. The above databases plus British Nursing Index (BNI) were searched from inception to July 2010 using the Ovid interface. The search strategy was as follows: exp depression/ OR exp depressive disorder/ OR exp depressive disorders/ OR exp major depression/ OR depressed.ti,ab. OR depression.ti,ab. OR depressive.ti,ab. OR dysthymi *.ti,ab. AND exp drugs, chinese herbal/ OR exp Medicine, Chinese Traditional/ OR exp Medicine, Oriental Traditional/ OR exp chinese drug/ OR exp chinese medicine/ OR exp chinese herb/ OR (exp medicinal herbs/ AND chinese.mp.) OR (chinese adj3 herb *) OR (chinese.ti,ab. AND herb *.ti,ab.). Cochrane CENTRAL was searched and a search was conducted of the Current Controlled Trials metaregister (http://www.controlled-trials.com/mrct/) to identify possible unpublished trials. The most recent search for trials was carried out in July 2011.

Systematic reviews were identified from the above searches plus searches of the Cochrane Register of Systematic Reviews and the Database of Abstracts of Reviews of Effects (DARE). Searches for systematic reviews continued up to July 2012.

Search results were screened for trials by two reviewers working independently using the following inclusion criteria.

- Participants: diagnosed with clinical depression using conventional or traditional Chinese criteria. Trials in which participants with different diagnoses were treated (i.e., depression or bipolar disorder) were included if outcomes were reported separately for patients with depression.
- Interventions: any intervention using Chinese herbal formulae (combinations of herbs) or single Chinese herbs.
- Comparison interventions: included placebo, no treatment, or any active treatment.
- Outcomes: improvement in depression (partial or complete alleviation of symptoms), as measured by validated outcome measure, clinical assessment of improvement, and participants' subjective experiences where reported.

Trials in which Chinese herbs were combined with other complementary therapies (i.e., Chinese herbs and acupuncture) were excluded. Studies on participants with depression associated with other conditions, for example, a medical/physical condition, menopause, premenstrual syndrome, or bipolar disorder were identified initially. However, as the herbal mixtures used may have had effects other than on depression, the main data analysis focused on those in depression not associated with another condition. Selections were compared and differences resolved by discussion.

Systematic reviews were identified by one researcher (KP) using the following inclusion criteria: reviews of Chinese herbal medicine in depression reported to be systematic and including methods for searching and selection of studies.

### 3.2. Data Extraction and Assessment of Methodological Quality

Key criteria were used for systematic reviews. These included focus, date of searches, sources, inclusion criteria, extraction and appraisal methods, number of trials included, method of synthesis of results, and conclusions. A data extraction sheet was designed to capture key data from trials and allow an accurate comparison of the studies selected. Translations were obtained for studies published in Chinese. Data collected included details of study design, participants, diagnostic criteria, interventions, outcomes measures, and results for the primary outcome measure. 

RCTs were evaluated using two rating scales: the Jadad criteria, use of which results in a score of between 0 and 5 based on 3 aspects of the methods: randomisation, blinding, and extent of followup [16]. This scale places great emphasis on blinding, which may not be feasible in trials involving mixtures of Chinese herbs. Therefore, a second more comprehensive scale was deemed necessary. The checklist proposed by Downs and Black [17] was considered appropriate. Previous reviews had found that there was good correlation with Jadad [16] and had independently validated its accuracy [18, 19]. Downs and Black [17] use a checklist of 27 questions which are intended to give broader overview of the methods used in RCTs and non-RCTs. The final question relating to power was adapted so that trial either scored 0 if power was not discussed or 1 if a power calculation was conducted and sufficient numbers recruited. The total possible score was, therefore, 28. Trials scoring 3 or more on the Jadad scale were considered to be at low risk of bias [16]. The approach taken by Malcomson et al. [20] was to consider trials scoring 50% or more on the Downs and Black scale as of good quality. In this paper, total scores for both scales and key risk of bias measures are reported.

## 4. Results

### 4.1. Previously Published Systematic Reviews

Six reports of 5 systematic reviews were identified [21–26]. Each of these focused on a specific aspect: trials in the Chinese literature [21], trials of the specific herbal formulae Xiao Yao San [22, 23], Free and Easy Wanderer Plus (a modified version of Xiao Yao San) [24], and Chaihu-Shugan-San [25], and trials of Chinese herbs as adjunctive treatment [26].

The first of these systematic reviews included 18 trials found by searching Chinese databases [21]. The trials involved a total of 1,260 patients and included trials in depression associated with various conditions such as cancer, stroke, and the menopause. The authors concluded that there was no evidence to support a beneficial effect in depression. The findings are difficult to interpret and the validity of the conclusions is unclear. Trials of combined treatment (acupuncture plus herbs) were included as were those in which treatment appeared to be aimed at the medical condition rather than depression. Assessment of quality involved assigning Jadad scores but these were not reported. A meta-analysis was conducted but the results of trials comparing Chinese herbs against placebo and those comparing Chinese herbs against active treatment appear to have been combined rather than reported separately.

A second systematic review focused specifically on Xiao Yao San (also known as Free and Easy Wanderer Powder or Rambling Powder) [22, 23]. This formula together with a modified version, Free and Easy Wanderer Plus (Jia wei Xiao Yao San or augmented Xiao Yao San) are two of the more frequently used formulae in Chinese medicine [27]. Two similar reviews have been published: the first, published in Chinese, included 32 trials (2,253 patients) and the second, published in English including 26 trials (1,837 patients) [22, 23]. The following comments relate to the second publication. The authors concluded that Xiao Yao San combined with antidepressants was more effective than antidepressants alone. Adverse effects were reported for antidepressants but not in relation to Xiao Yao San. The majority of trials were found to be of poor methodological quality. The conclusions also need to be interpreted in the context of potential publication bias as revealed by a funnel plot which demonstrated significant asymmetry. There was also considerable variation between the formulae used in the trials: only 8 trials used the traditional formula for Xiao Yao San, the remaining trials using a modification of the original formula. The total number of herbs used in each trial varied between 7 and 17.

The third review which was also published in 2011 addressed trials of Free and Easy Wanderer Plus [24]. Searches of Chinese and Western databases were carried out and 14 trials selected, all conducted in China and 9 of which included participants with major depression. Only trials scoring at least 3 on the Jadad scale were included, which had resulted in a further 50 trials being excluded from the meta-analysis. The authors concluded that the herbal formula may be effective in depression, may enhance conventional antidepressants, and may have a better safety profile than standard antidepressants.

In the first of the reviews published in 2012, English and Chinese databases were searched for RCTs in which Chinese herbs were used in combination with antidepressants [26]. Methods appeared rigorous and results presented as weighted mean difference (WMD) based on Hamilton rating scale for depression (HAM-D) scores. Seven RCTs involving 576 participants were identified. Meta-analysis indicated that integrated traditional and Western medicine based on syndrome differentiation produced a greater reduction in mean HAM-D scores than Western medicine alone (WMD −2.39 95% CI −2.96, −1.83). No serious adverse effects were reported for combined treatment, but all trials were evaluated as being at risk of bias or the risk of bias was unclear.

The fifth systematic review was also published in 2012 [25]. A range of Western and Chinese databases were searched for trials of the formula, Chaihu-Shugan-San in depression, and papers were selected by two researchers. Ten RCTs involving 835 subjects were included. Meta-analyses based on 6 RCTs indicated that Chaihu-Shugan-San in combination with various selective serotonin reuptake inhibitors (SSRIs) was more effective that antidepressant drugs alone based on HAM-D scores (WMD = −3.56; 95% CI −5.09 to −2.03). Based on two trials, Chaihu-Shugan-San as monotherapy was also more effective than antidepressants in improving depressive symptoms (WMD = −3.09; 95% CI −5.13 to −1.06). No serious adverse events were reported. As with previous reviews, all studies were judged to be of poor methodological quality and at risk of bias.

No systematic reviews were found in which Western databases were searched for trials of all Chinese herbs or herbal formulae in depression. In the systematic reviews described above, either only Chinese databases were searched or the review only included trials of one specific formula. A systematic review was conducted to address this gap (supplementary systematic review) and the results are described below.

### 4.2. Supplementary Systematic Review

**Figure 1:**
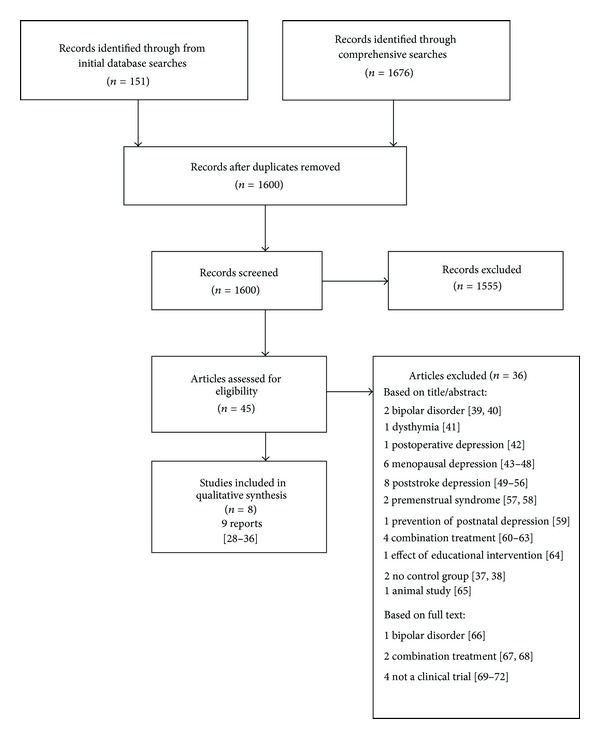
Flowchart showing selection process.

A total of 1600 citations were retrieved (151 from the initial search; 1676 from the comprehensive searches of which 1449 remained after removing duplicates). No unpublished trials were identified from the trials register. Forty-five potentially relevant citations were selected from these. Trials were identified of Chinese herbs in the treatment of depression, in perimenopausal depression, poststroke depression and depression associated with other medical and psychiatric conditions, and in the prevention of postnatal depression. For depression as the primary diagnosis, no nonrandomised controlled trials were located but two uncontrolled studies were located: one in which 40 cases of “melancholia” were treated with a formula called modified Wen Dan Tang [28] and the other in which 20 patients with prolonged partial remitted major depressive disorder associated with fatigue or loss of energy were treated with Japanese formulae comparable to the two Chinese formulae: Liu Wei Di Huang Wan and Bai Wei Di Huang Wan [29]. The RCTs [30–38] are discussed in the following section, and Figure 1 shows a summary of the selection process and the excluded studies [28, 29, 39–72]. 

#### 4.2.1. Summary of Trials Located

A total of nine reports of controlled trials of Chinese herbs in depression were identified [30–38] (see Table 1 for a summary of the trials). Two reports appeared to be of different outcomes from one trial [31, 32]. In all cases, the trials were described as RCTs. Participants had been diagnosed with depression by various means: using the Diagnostic and Statistical Manual of Mental Disorders (DSM-IV) criteria, the Chinese Classification of Mental Disorders (CCMD-3) criteria, CCMD-3 plus the International Classification of Diseases (ICD-10), and Chinese medicine diagnostic methods. In 7 trials, the inclusion criteria included a minimum score on the HAM-D scale. The minimum scores required were generally between 17 and 20 reflecting mild-to-moderate depression. However, the mean scores were higher than this in some trials (and over 30 in two trials), suggesting that more severe cases of depression were included. Two trials were placebo-controlled [34, 38] and one was a double-placebo-controlled trial [31, 32]. Two trials compared a Chinese herbal formula with the antidepressants: fluoxetine and maprotiline, respectively [30, 33]. In two trials, Chinese herbs combined with an antidepressant were compared against antidepressant alone [35, 36]. In both cases, the Chinese herbs were combined with a tricyclic antidepressant but in Yang et al.'s study [35], the comparison was against an SSRI. The final trial was a 3-arm trial comparing two different Chinese herbal formulae combined with fluoxetine against fluoxetine alone [37].

#### 4.2.2. Setting, Size, and Duration

All trials had been conducted in China and patients appeared to have been recruited from a variety of sources. The authors were based at hospitals or medical colleges and one could assume that this is where the trials were conducted but this was not explicitly stated in all cases. The number of subjects recruited ranged from 60 to 164 (total 756) and the treatment duration ranged between 30 days and 12 weeks. None of the trials conducted a later followup to ascertain whether changes were maintained or whether remission was only temporary.

#### 4.2.3. Chinese Herbs Investigated

All trials involved the use of Chinese herbal formulae rather than a single herb. A variety of different Chinese herbal formulae were investigated. Of the herbs included in the formulae, only one, *Hypericum *(St John's wort) has documented anti-depressive activity [73]. This herb was included in the formula used in one trial. According to the same reference source, four of the herbs used have potential sedative or anxiolytic properties. Preliminary evidence has also been reported in studies from China on relevant effects of several herbs included Xiao Yao San [24].

#### 4.2.4. Outcome Measures

The effects of the herbal formulae were assessed in 7 of the trials by comparing HAM-D scores before and after treatment. Rating scales used in addition to HAM-D included the Self-Rating Anxiety Scale (SAS), Self-Rating Depression Scale (SDS), Montgomery-Asberg Depression Rating Scale (MADRS), Clinical Global Impression (CGI), TCM syndrome and symptom differentiation (TCM-SSD), and Treatment Emergent Symptom Scale (TESS). In one trial, response was only measured by clinical assessment of symptom improvement or resolution [30].

#### 4.2.5. Reporting of Adverse Events

All but two trials reported types of adverse events by intervention. Zhang et al. [38] and Sun et al. [34] found there to be no statistical difference between incidence of adverse events in the Chinese herb group and the placebo group. A significant difference was reported between treatment with a Chinese herbal mixture with or without an antidepressant compared with the antidepressant alone, with the herbal mixture apparently causing reduced adverse event rates [31–33, 36, 37]. In the other studies, a formal statistical comparison was not carried out and adverse events simply reported by group. 

#### 4.2.6. Overall Quality of Methods and Reporting

All studies were described as randomised but several did not describe how randomisation had been carried out. The Jadad assessment also revealed that an effective process for blinding (or masking) treatment was only described in three trials. From the assessment based on the Downs and Black checklist, the following items were found to be reported in few, if any, studies: how recruitment to the study was carried out, whether intervention and control groups were wellmatched at baseline, which statistical tests were used, whether compliance was reliable, and whether the study had sufficient power.

The mean Jadad score was 2.4 (out of 5) and 3 trials scored more than 3. The mean adapted Downs and Black's score was 17 (out of 28). Only two trials scored more than 20 (both of which also scored 4 on the Jadad scale). The scores on each of the aspects addressed by the Downs and Blacks checklist varied from 5 to 11 (out of 11) for reporting, 0 to 1 (out of 3) for external validity, 1 to 6 (out of 7) for internal validity (bias), and 3 to 6 (out of 6) for internal validity (confounding). No trial discussed power or provided a rationale for the number of participants recruited. Based on the risk of bias assessment, trials were either at risk of bias or the overall risk of bias was unclear.

#### 4.2.7. Pooling of Results

The herbal formulae and control treatments used, trial duration, and trial design all differed to such an extent that it was not possible to pool data to present any meaningful statistics. Where changes in depression scores were measured, clinically significant reductions were reported within groups with active treatment, either Chinese herbs or antidepressants. However, between-group comparisons suggested that Chinese herbs were more effective than antidepressants [30], were comparable to antidepressants [31–33], did not increase effectiveness but reduced adverse effects or relapse rates when used as additive therapy with antidepressants [35–37], or were more effective than placebo [34, 38].

### 4.3. Comparison with the Other Systematic Reviews

Only three trials were located that had not been included in the previous reviews [30, 34, 36]. Two of these assessed the formula, Shugan Jieyu and the third, modified Xiao Yao San. Two trials were included in the previous review focusing on the Chinese literature [33, 37], three were included in the systematic review of Xiao Yao San formula [31, 32, 35, 37] and two were in the Free and Easy Wanderer Plus review [31, 38]. None of the trials were included in the reviews of Chaihu-Shugan-San or Chinese herb/antidepressant combination treatment. A comparison of the systematic reviews is presented in Table 2. In terms of methodology and reporting, the current review found similar issues to the previous systematic reviews. These included lack of reporting of whether groups were matched on baseline characteristics, allocation concealment or blinding of assessors. Little or no detail was provided on dropout and intention-to-treat analysis. These address issues related to internal validity. As described above, the current review also revealed issues affecting external validity in that the process of recruitment of participants and the specific location of the trial were not reported in most trials. 

An evaluation of the results of the meta-analyses and the strength and quality of the supporting evidence was conducted. The results are presented in Table 3. This demonstrates that the overall evidence was generally of low quality and even moderate evidence was compromised by aspects such as the heterogeneity of the interventions and diagnoses. 

## 5. Discussion

This paper provides an overall picture of the current evidence base for Chinese herbal medicine in the treatment of depression. Five published systematic reviews were located. A supplementary systematic review to address a gap in the coverage located eight trials, of which three had not been included in previous systematic reviews.

Positive results were reported almost universally. These included one or more of the following: greater anti-depressive effects than placebo, equivalent effects to antidepressants, less problems with adverse effects than antidepressants, or reduction of the adverse effects caused by antidepressants when used in combination. In trials comparing Chinese herbs against antidepressants, lack of power calculations means that it is unclear whether a lack of difference between the Chinese herbal formula and the antidepressant is due to a true difference or simply a trial that was underpowered to detect a difference. Of the RCTs found, five scored less than 3 on the Jadad scale which suggests bias may have been introduced. In several cases, the low score was due to blinding being impossible but lack of information on withdrawals and dropouts was also a problem. This finding correlates with previous reviews, which also reported low scores on Jadad or unclear or high risk of bias for many of the included trials. In fact, based on risk of bias assessments, all trials were either at risk of bias or the extent of possible bias was unclear.

All 8 trials were conducted in China and the methods used in most of the trials were similar. Diagnosis was using conventional diagnostic frameworks and the response measured using the HAM-D instrument. Patients appear to have been recruited via hospitals in most cases but the exact process of recruiting patients was not reported in any trial. Thus, it is difficult to assess whether the patients selected were representative of the population from which they were selected. Similarly, trials were reported to be randomised but it is difficult to judge whether allocation was effectively concealed. In fact, the limited reporting precludes an accurate assessment of the methods and, therefore, the reliability of the results. Again, this is a similar finding to those of the other systematic reviews in this area.

Herbs were used in combination and at least 6 different herbs appear to have been included in many of the formulae. Several of the herbs have sedative or anxiolytic potential activity which may be beneficial in depressed patients [39]. The remaining herbs have a range of uses and actions and it becomes more apparent why a formula such as *Xiao Yao San* with or without modifications might be used widely for a range of conditions. It is also possible that using these herbs in combination may produce effects that would not be achieved with each herb alone. 

Chinese herbal mixtures are supplied as the dried plant parts, pills or capsules, or in powder form [27]. Different parts of the plant are used and preparation of the dose may entail processes such as decoction which involves boiling. The effect of these processes on the activities of the component herbs is difficult to predict. In terms of trial design, blinding or masking of the patient is, in many cases, impossible. Even if pills or capsules are prepared, unblinding may take place due to the smell of the herbs. This obviously may introduce some bias on the part of the patient which is particularly relevant in depression where the main outcome measure is based on self-report. Blinding of assessors may limit the extent of bias somewhat but was not reported in the trials included in this paper, nor have other reviewers found consistent reporting of blinding.

Two systematic reviews have focused on the formula Xiao Yao San or a modification of this [23, 24]. In practice, this formula forms the basis for an array of modified formulae. From the Chinese medicine perspective, these are all based on the core formula, with additional herbs added to address specific problems. They are referred to as modified Xiao Yao San or Jia wei Xiao Yao San (san means powder while wan means pill). Thus, two preparations may have a similar name but contain different herbs [27]. This causes potential problems in interpreting the results of trials using these formulae. It also causes problems in practice in the reporting of adverse events and checking for interactions. The formulae used are considered safe by TCM practitioners based upon experiential evidence but there is obviously a potential for interactions and adverse effects. Some insight into the more frequent adverse effects is revealed by the results of these trials but the small size of the trials means that less common adverse effects may not have been encountered.

The issue of diagnostic frameworks is also worth considering when assessing the relevance of these trials to practice. Chinese medicine recognises patterns of signs and symptoms and diagnoses that do not fit with a Western framework [27]. People who might be diagnosed in Western medicine with depression may receive different Chinese medical diagnoses depending on the overall pattern. Virtually all the trials have used standardised Western-based diagnostic frameworks. The advantage is more ready interpretation in a Western context but the primary problem is whether the trials reflect usual Chinese medicine practice. Related to this is the fact that virtually all the research has to date been conducted in China so it is difficult to translate the results into a Western healthcare context.

None of the trials reviewed here conducted a followup at a later date to ascertain duration of antidepressant effects after the herbal treatment. The longest duration was 12 weeks. Without this information it is impossible to establish whether the herbal medicine had a temporary effect or whether its effects were long lasting or to be able to calculate relapse rates. National guidelines on the management of depression [74] state that drugs administered for depression should be continued for 6 months after the last episode to avoid relapse. If the same is true of herbal formulas, then a long-term trial would be necessary to test long-term benefits of herbal medication.

Analysis of the overall strength and quality of the evidence from the various systematic reviews revealed a number of aspects which compromise firm conclusions on the effectiveness of Chinese herbal formulae in the treatment of depression.

### 5.1. Limitations of This Review

Only systematic reviews indexed in Western databases were included. However, the Chinese literature was addressed to some extent in that each of these systematic reviews involved searches of Chinese databases. In the supplementary review, all except one of the trials included were originally published in Chinese. Therefore, the data presented and quality assessments were based on translations rather than the original reports.

## 6. Conclusions

Overall, this paper has provided an insight into the research that has been conducted on Chinese herbs in depression. The intention of this paper was to assess the effectiveness of Chinese herbal medicine treatments for depression. Positive results were reported in all the trials identified and in all but one of the systematic reviews. These results included no significant differences when compared with medication, greater effect than medication or placebo, and reduction in adverse event rates when used as additive therapy. However, because of limitations in the strength and quality of the evidence, it has not proved possible to either fully substantiate or disprove claims of effectiveness of Chinese herbal medicine in the management of depression. Limitations in reporting and in methodology, different control interventions, and use of varied formulae precluded any reliable conclusions. In addition, virtually all trials located were for 12 weeks or less so that it is unclear whether reported effects were sustained in the longer term. Adverse effects were reported but trials were generally small and the preparations used varied so that further evidence on safety would be required for firm conclusions.

It is clear that a large number of trials of Chinese herbs in depression have been conducted. Based on the findings of the supplementary review, only a small proportion of these are currently listed in Western databases. Similarly, in the case of the systematic reviews, it is also possible that further systematic reviews are available in the Chinese literature. However, research to change or support practice in Western contexts needs to be accessible, thus supporting the rationale for focusing on evidence found in the Western literature while drawing attention to the wider literature on this topic.

Conclusions of potentially beneficial effects, particularly for the formula *Xiao Yao San* and modified versions of this formula, need to be interpreted in the light of limitations related to reporting and methods but also because of the variation in the combinations of herbs used.

Nevertheless, this overview of the evidence indicates that well-designed trials in a Western context may be worth pursuing. These will require decisions such as which framework is used to diagnose depression, whether any flexibility is allowed in the prescribing of Chinese herbs and the most appropriate comparison intervention. It is possible that pragmatic trials comparing overall care via different systems will be most informative. Reporting does need to be addressed because it affects how an RCT is judged, particularly when scoring systems such as Jadad are used to assess quality. Assessment of preference and expectations will also be important in interpreting the results where disguising the treatment is not possible.

## Figures and Tables

**Table 1:** Summary of the RCTs included in the Western literature (unique studies shown in bold).

Study	Setting and duration	Sample Size	Diagnosis and severity	CHM treatment (no. treated)	Control Treatment (no. treated)	Outcome measures	Results*	Adverse events	Jadad, DB and ROB*
**Li et al. 2006 [30]** **(English)**	**China (hospital outpatients)** **30 days**	**164**	**Senile depression** **TCM diagnostic criteria**	**Shugan Jieyu Yin decoction 100 mL twice daily (84)**	**FLU 20 mg twice daily** **(80)**	**Clinical assessment based on TCM criteria**	**Cure/markedly relieved/improved/failed:** **CHM 40% 27% 24% 8% ** **FLU 3% 24% 38% 36% ** **Significant difference in total effective rate (92% versus 64%; *P *** < **0.01)**	**Not reported**	**J:1** **DB:12** **ROB:** **Unclear** **Unclear** **High** **High** **Unclear** **Unclear**

Luo et al. 2006* [31] (Chinese) and Li et al. 2007* [32] (Chinese)	China (hospital outpatients) 6 weeks	66	Depression CCMD-3 ICD-10 TCM diagnosis HAM-D > 20 SAS and SDS > 53	Danzhi Xiaoyao Powder 12 g twice per day plus placebo (34)	MAP 25 mg per day increasing to max. 250 mg per day plus placebo (32)	HAM-D SDS SAS SERS (As above plus neuro-immuno-endocrine outcomes for Li et al. 2007 [32])	Mean reduction in HAM-D: CHM: 35.90 to 10.62 MAP: 34.16 to 8.29 (reported as: CHM: 35.59 to 11.22 MAP: 34.16 to 8.77 in Li et al. 2007 [32]) Significant reduction within both groups (*P* < 0.01), NS difference between groups	CHM: 9 reports MAP: 91 reports significant difference (*P* < 0.05)	J:4 DB:17 ROB: Low Low Low Low Unclear Unclear

Shen et al. 2004 [33] (Chinese)	China (6 centres) 6 weeks	60* *3 cases lost	Depression CCMD-3 ICD-10 HAM-D 20+ SDS 50+	Jieyu 60 pills 3 times daily (28)	MAP 25 mg daily increasing to 100–250 mg (29)	HAM-D SDS SAS CGI ARS	Mean reduction in HAM-D: CHM 35.93 to 9.71 MAP 38.48 to 8.90 Significant reduction within both groups (*P* < 0.01), NS difference between groups	MAP: range of reported adverse effects CHM: mild headache, fatigue significant difference (*P* < 0.01)	J:2 DB:15 ROB: Low Unclear Unclear Unclear Unclear Unclear

**Sun et al. 2009 [34]** **(Chinese)**	**China** **(multicentre)** **6 weeks**	**120**	**Depression** **CCMD-3** **HAM-D 17–28** **TCM diagnostic criteria**	**Shugan Jieyu capsule twice daily (80)**	**Placebo capsule twice daily (40)**	**HAM-D** **CGI** **STCM score**	**Response rates based on HAM-D: CHM 68%, placebo 29% ** **based on STCM: CHM 59%, placebo 23.7%** **Significant difference between groups (*P *** **<** **0.01) **	**CHM: 30.4% (24/79)Placebo 23.1% (9/39). ** **No serious adverse events. ** **NS difference **	**J:4** **DB:20** **ROB:** **Low** **Unclear** **Low** **Low** **Low** **Unclear**

Yang et al. 2007 [35] (Chinese)	China 12 weeks	64	Depression CCMD-3 HAM-D >18	Modified Xiaoyao 9 g pill twice daily plus AMI 25–150 mg per day (32)	FLU 20–40 mg per day (32)	HAM-D Clinical assessment	Mean reduction in HAM-D: CHM + AMI 29.36 to 4.35 FLU: 30.18 to 4.18 NS difference between groups. Relapse rates lower in CHM + AMI group (3 versus 14 cases)	CHM + AMI: 12 reports FLU: 12 reports	J:2 DB:14 ROB: Low Unclear Unclear Unclear Unclear Unclear

**Yu et al. 2007 [36]** **(Chinese)**	**China (hospital)** **8 weeks**	**105**	**Depression** **CCMD-3** **HAM-D 17+** **TCM diagnostic criteria**	**Modified Xiaoyao decoction** **plus CLOM 25**–**50 mg** **per day (53)**	**CLOM 75**–**225 mg** **per day (52)**	**HAM-D** **CGI-SI** **Clinical assessment**	**Mean reduction in HAM-D:** **CHM** **+** **CLOM: 28.48 to 7.89 ** **CLOM: 28.22 to 7.91** **Significant reduction within both groups (*P *** < **0.01), NS difference between groups**	**CHM** **+** **CLOM:** **9 reports ** **CLOM: 40 reports ** **Significant difference (*P *** < **0.01)**	**J:0** **DB:15** **ROB:** **High** **High** **High** **High** **Unclear** **Unclear**

Zhang et al. 2006 [37] (Chinese)	China (hospital inpatients and outpatients) 6 weeks	90	Senile depression CCMD-3 HAM-D >18	Xiaoyao 8 pills 3 times per day plus FLU 20 mg (30)	Sanpu xinnao xin 2 pills 3 times per day FLU 20 mg (31) FLU 20 mg–40 mg only (29)	HAM-D TESS	Mean reduction in HAM-D: XY + FLU: 28.58 to 10.29 SX + FLU: 26.41 to 10.45 FLU: 27.76 to 10.26 NS difference between groups	XY + FLU: 15 reports SX + FLU:14 reports FLU: 26 reports Significant difference (*P* < 0.01)	J:2 DB:17 ROB: Unclear Unclear Unclear Unclear Unclear Unclear

Zhang et al. 2007 [38] (English)	China (7 sites) 12 weeks	87 (plus 62 bipolar)	Depression DSM-IV HAM-D 18+	Free and Easy Wanderer Plus* (Jia Wei Xiao Yao San) 36 g/day in 3 doses (49)	Placebo tablets (38)	HAM-D MADRS CGI-S	Mean reduction in HAM-D: CHM: 23.6 to 8.1 Placebo: 24.0 to 13.1 Significant difference between groups (*P* < 0.05)	Most frequent: dizziness, headache NS difference	J:4 DB:23 ROB: Low Unclear Low Low Low Unclear

*Note: HAM-D scores are baseline and final mean scores. ARS: Asberg Rating Scale; CCMD: Chinese Classification of Mental Disorders; CGI-S: Clinical Global Impression-Severity scale; D and B: Downs and Black; HAM-D: Hamilton Rating Scale for depression; J: Jadad; MADRS: Montgomery-Asberg Depression Scale; NS: non-significant; SAS: Self rating Anxiety Scale; SDS: Zung's Self rating Depression Scale; SERS: Side Effect Rating Scale; STCM: Symptom of traditional Chinese medicine; TCM: traditional Chinese medicine; TESS: Treatment Emergent Symptom Scale. Drugs: AMI: amitriptyline; CLOM: clomipramine; FLU: fluoxetine; MAP: maprotiline. *ROB (Risk of bias) was reported random sequence generation, allocation concealment, blinding of participants and personnel, blinding of outcome assessment, incomplete outcome data, selective reporting.

**Table 2:** Comparison of systematic reviews.

	Kou and Chen 2012 [26]	Qin et al. 2011 [22]	Wang et al. 2012 [25]	Zhang et al. 2012 [23]	Zhao et al. 2009 [21]	Current SR
Herbs included	Chinese herbs combined with ADs	Free and Easy Wanderer Plus (and modifications)	Chaihu-Shugan-San	Xiao Yao San (and modifications)	Chinese herbs	Chinese herbs
Comparison	AD only	AD, placebo	AD	AD	Various treatments	AD, placebo
Databases searched	Chinese and Western	Chinese and Western	Chinese and Western	Chinese and Western	Chinese	Western
Date of searches	March 2010	December 2010	December 2010	November 2009	July 2008	July 2011
Diagnostic criteria	Not restricted	Not restricted	CCMD/DSM/ICD	Not restricted	“Western criteria”	Not restricted
Types of trials included	RCTs	RCTs	RCTs	RCTs	RCTs and quasi-RCTs	RCTs
Number of trials (participants)	7 (576)	14 (1224)	10 (835)	26 (1837)	18 (1260)	8 (756)
Outcome measures	HAM-D	HAM-D	HAM-D	Clinical effect, HAM-D, SDS	HAM-D, SDS	Various
Extraction and Assessment process	not reported	2 reviewers independently	2 reviewers independently	2 reviewers independently	not reported	2 reviewers independently
Evaluation method	Risk of bias	Jadad plus 3 criteria	Modified Jadad	Risk of bias	Jadad	DB, Jadad, ROB
Bias/quality of trials	Unclear/high risk	All scored 3+	All scored <4	Unclear/high risk	All scored 1-2	Unclear/high risk
Meta-analysis results						
Herb versus placebo	—	OR 9.40 [5.57,15.89]	—	—	Unclear	—
Herb + AD versus AD	WMD −2.39 [−2.96, −1.83]	OR 1.75 [1.26, 2.44]	WMD −3.56 [−5.09, −2.03]	WMD −0.51 [−0.71, −0.31]	—	—
Herb versus AD	—	OR 1.09 [0.60, 1.98]	WMD −3.09 [−5.13, −1.06]	WMD 0.43 [−2.14, 2.99]	—	—
Overall results	Positive	Positive	Positive	Positive	Negative*	Inconclusive

Key: AD: antidepressants, DB: Downs and Black, ROB: risk of bias, WMD: weighted mean difference, *results unclear.

**Table 3:** Summary of results and supporting evidence (based on meta-analyses).

Outcome	Intervention	Control	Result (95% CI)	Evidence (participants)	Quality* (comments)
HAM-D score	Chinese herbs + ADs	ADs alone	WMD −2.39 [−2.96, −1.83]	7 RCTs (576)	Low (trials unclear/high risk of bias, varied herbs)
			WMD −3.56 [−5.09, −2.03]	6 RCTs (506)	Low (trials low quality, heterogeneity)
			WMD −0.51 [−0.71, −0.31]	14 RCTs (921)	Low (trials unclear/high risk of bias, heterogeneity)
			OR** 1.75 [1.26, 2.44]	8 RCTs (648)	Low/moderate (varied diagnoses)
	Chinese herbs	ADs	WMD −3.09 [−5.13, −1.06]	2 RCTs (164)	Low (trials high risk of bias, heterogeneity)
			WMD 0.43 [−2.14, 2.99]	3 RCTs (NR)	Low (trials unclear/high risk of bias, publication bias)
			OR** 1.09 [0.60, 1.98]	4 RCTs (250)	Low/moderate (varied diagnoses)
TESS score	Chinese herbs	Placebo	OR** 9.40 [5.57, 15.89]	3 RCTs (321)	Low (heterogeneity)
	Chinese herbs/ADs	ADs alone	WMD −2.51 [−3.18, −1.84]	4 RCTs (263)	Low (heterogeneity)
	Chinese herbs	ADs	WMD −1.86 [−2.57, −1.15]	1 RCT (60)	Very low (single trial, high risk of bias)

TESS: Treatment emergent symptoms and side effects; NR: not reported; *Overall quality of the evidence was assessed based on reported quality/potential bias in RCTs, heterogeneity, publication bias, consistency of interventions and diagnoses. **Odds ratios were based on a decrease of at least 50% in HAM-D scores.
